# Molecular epidemiology and drug resistance prevalence of strains from newly diagnosed HIV-1 patients in Northern Greece during 2009-2010

**DOI:** 10.1186/1742-4690-9-S1-P30

**Published:** 2012-05-25

**Authors:** Zoe Antoniadou, Ioanna Kousiappa, Johana Hezka, Lemonia Skoura, Simeon Metallidis, Pavlos Nikolaidis, Nicolaos Malisiovas, Leondios G Kostrikis

**Affiliations:** 1 University of Cyprus, Nicosia, Cyprus

## Introduction

As part of a continuing effort to monitor the molecular epidemiology of HIV-1 in northern Greece, in this study we determined the genetic diversity and the prevalence of drug resistance transmission among HIV-1 strains isolated from 94 newly-diagnosed untreated consenting patients in the period 2009 to 2010.

## Materials and methods

Peripheral blood mononuclear cells (PBMC’s) and plasma were collected from the patients at the AIDS National Reference Laboratory of Northern Greece. Plasma RNA encoding partial pol(protease and reverse transcriptase) was amplified using TRUGENE HIV-1 genotyping assay (Siemens). Antiretroviral drug resistance prevalence was estimated by using the HIVseq program (HIV Drug Resistance Database, Stanford University). PBMC’s DNA encoding partial env(gp120) V3-loop region was amplified by nested PCR and sequenced using an in-house tropism assay at the Department of Biological Sciences, University of Cyprus. Phylogenetic analysis was performed and trees were constructed for each region by using neighbor-joining with Kimura two-parameter method by means of MEGA v5.0 software.

## Results

The media age of patients is 32 years with the percentage of male infection at 89.4%. The main risk groups were homosexual contact (73.4%) and heterosexual contact (17.0%). The phylogenetic analysis indicated B and A1 as the dominant subtypes, 47.8% and 41,5% respectively, followed by subtype C, CRF02_AG (3,2% each), CRF04_cpx (2,1%) and subtypes F1 and G (1,1% each). Twenty-one clusters showed epidemiologically linked HIV-1 patients. Resistance mutations to protease inhibitors were found in three individuals, while high resistance associated mutations to reverse transcriptase inhibitors (NRTI’s/NNRTI’s) were shown in sixteen patients. Tropism testing indicated most of the HIV-1 strains as R5-tropic (96%).

## Conclusions

These newly found data demonstrate a heterogeneous epidemiological status of HIV-1 in northern Greece during 2009-2010, with subtype B and A1 being the dominant subtypes in relation to the other subtypes. The prevalence of antiretroviral resistance mutations is high among the newly diagnosed untreated patients (17%) in comparison with other European countries (10%).

